# Prevalence of common mental disorders among sugarcane workers

**DOI:** 10.11606/S1518-8787.2017051007140

**Published:** 2017-12-04

**Authors:** Polyana Felipe Ferreira da Costa, Solange Laurentino dos Santos, Marcelo Saturnino da Silva, Idê Gomes Dantas Gurgel

**Affiliations:** I Fundação Oswaldo Cruz. Instituto Aggeu Magalhães. Programa de Pós-Graduação em Saúde Pública. Recife, PE, Brasil; II Universidade Federal de Pernambuco. Departamento de Medicina Social. Recife, PE, Brasil; III Universidade Estadual da Paraíba. Departamento de Educação. Guarabira, PB, Brasil; IV Fundação Oswaldo Cruz. Instituto Aggeu Magalhães. Departamento de Saúde Coletiva. Recife, PE, Brasil

**Keywords:** Rural Health, Transients and Migrants, Mental Disorders, epidemiology, Risk Factors, Working Conditions, Cross-Sectional Studies, Saúde da População Rural, Migrantes, Transtornos Mentais, epidemiología, Fatores de Risco, Condições de Trabalho, Estudos Transversais

## Abstract

**OBJECTIVE:**

To estimate the prevalence of common mental disorders and to analyze the associated factors in migrant and sugarcane workers.

**METHODS:**

This is a cross-sectional study carried out with 110 workers. Common mental disorders were evaluated using the Self-Reporting Questionnaire (SRQ-20), and sociodemographic, occupational, and lifestyle variables were studied. The CAGE questionnaire was used to detect the abuse of alcoholic beverages.

**RESULTS:**

The prevalence of common mental disorders affected 40% of the workers and the association showed statistical significance for the positive result of the CAGE test, sickness, absence from work, and medical care during the harvest period.

**CONCLUSIONS:**

The suspected cases of problem drinkers and the control mechanisms used by the mill for workers who miss work or become ill are factors that can cause common mental disorders.

## RESUMO

**OBJETIVO:**

Estimar a prevalência de transtornos mentais comuns e analisar os fatores associados em trabalhadores migrantes e canavieiros.

**MÉTODOS:**

Estudo transversal realizado com 110 trabalhadores. Os transtornos mentais comuns foram avaliados por meio do *Self-Reporting Questionnaire* (SRQ-20) e foram estudadas variáveis sociodemográficas, ocupacionais e de estilo de vida. Para detectar o uso abusivo de bebidas alcoólicas, foi utilizado o questionário CAGE.

**RESULTADOS:**

A prevalência de transtornos mentais comuns afetou 40% dos trabalhadores e a associação mostrou significância estatística para a positividade no teste CAGE, adoecimento, falta ao trabalho e atendimento médico durante o período da safra.

**CONCLUSÕES:**

A suspeita de casos de bebedores-problemas e os mecanismos de controle utilizados pela usina para os trabalhadores que faltam ou adoecem são fatores que podem causar transtornos mentais comuns.

## INTRODUCTION

Common mental disorders (CMD) are characterized by symptoms such as fatigue, irritability, forgetfulness, difficulty concentrating, and somatic complaints^1^. Their identification does not result in any diagnosis, only the survey of possible predispositions for mental illness^2^. The current epidemiological basis is characterized by studies aimed at the quantitative investigation of specific categories of workers, in order to evaluate the actual working conditions, the characteristics of the organizations, and the profile of sickness of workers, seeking to evaluate associations between these conditions and the observed labor characteristics and sickness^3^.

According to Ludermir and Melo Filho^4^, psychiatric epidemiology has identified an association between CMD and variables related to living conditions and occupational structure. Thus, it is a serious public health problem and has relevant economic impacts, because of the demands generated by health services and absenteeism at work^a^.

The work in the sugarcane fields requires physical strength and manual skills with the working tool – the machete. The worker needs, among other things, to obey the prescribed rhythm of work, to follow the intensity of the production, and to overcome the hazards and unhealthy conditions of this type of work^5^. Manual labor in sugarcane harvesting poses risks to the health of workers because of the intense heat, constant solar radiation, dust from the soil, and the presence of venomous animals. There is also a marked risk of work accidents because of the handling of the machete^6^.

It is known that, in addition to the risk situations arising from the labor relations and process, most subjects, as migrants, also experience situations of vulnerability in living spaces, paths, and in their own relations with the “natives” in work or leisure spaces^7^.

Alcoholism among rural workers is a response to chronic work stress that emerges when the coping strategies that the individual habitually employs to manage stress fail. Behaving as a mediating variable between perceived stress and its consequences, alcoholism is a habit typically manifested with psychological symptoms, being closely related to job dissatisfaction^8^.

In this context, we highlight the reality that is present in the micro-region of Pajeú, in the State of Pernambuco, and parts of the micro-region of Serra da Teixeira, in the State of Paraíba, where a large portion of the economically active population has becoming part of the seasonal migration to work especially on the harvest of sugarcane in the micro-region of São José do Rio Preto, in the State of São Paulo^9^ ^,^ ^10^.

In this article, we aim to identify the prevalence of CMD and analyze the associated factors in migrant and sugarcane workers.

## METHODS

This is a descriptive, cross-sectional study carried out with migrant workers from Paraíba and Pernambuco, who work in the sugarcane harvest in the region of São José do Rio Preto, State of São Paulo, Brazil, specifically in the municipalities of Novo Horizonte and Mendonça. These municipalities have industrial units of the same company of the sugar and ethanol sector, which became known as one of the main mills that recruits the labor directly in the region of origin of these workers.

Data from the Demographic Census of the Brazilian Institute of Geography and Statistics (IBGE, 2010) show that approximately 4.9% and 6.5% of the population living in the municipalities of Novo Horizonte and Mendonça, respectively, were from the Northeast region in the results of the migration sample^b^.

The reference population contained all workers employed at the studied mill (110 sugarcane workers), during the research period (July to September 2014), and who were from municipalities in Paraíba and Pernambuco. The number of participants (n = 110) in the study corresponded to all workers hired in the current harvest.

For data collection, we used a questionnaire with sociodemographic and occupational data and the Self-Reporting Questionnaire (SRQ-20). The SRQ-20 is an instrument that evaluates the occurrence of CMD, developed by Harding et al.^11^ and validated in Brazil by Mari and Willians in 1986, with sensitivity of 85% and specificity of 80%. A research conducted in Pernambuco, with psychiatric interview as gold standard, obtained sensitivity of 62% and specificity of 80%^12^.

The SRQ-20 has 20 items on physical and psychic symptoms, with a range of dichotomous answers (yes or no) for the detection of common mental disorders. Each affirmative answer has a score of 1 for the final score from the sum of these values. The scores obtained are related to the probability of presence of non-psychotic disorder, ranging from 0 (no probability) to 20 (extreme probability). The cutoff points are 7/8, regardless of sex, as described by Gonçalves et al.^13^; that is, the answers that present score ≥ 7 were considered as indicators of possible CMD.

The study investigated the different aspects of the workers’ lives, such as sociodemographic conditions (age, education level, marital status, number of children), housing conditions (type of housing and relationship between worker and other residents), work (number of harvests worked, average monthly wage in the last harvest, work-related morbidity, absence from work, and medical care during the harvest), and lifestyle (use of alcoholic beverages, tobacco, and other drugs). The CAGE questionnaire was used to detect the abuse of alcoholic beverages and it was included in the lifestyle category.

The CAGE test is also a standardized questionnaire consisting of four questions. Its denomination is derived from the keywords of each question: 1) have you ever felt you needed to *Cut down* on your drinking?; 2) have people *Annoyed* you by criticizing your drinking?; 3) have you ever felt *Guilty* about drinking?; 4) have you ever felt you needed a drink first thing in the morning to steady your nerves or to get rid of a hangover? *(Eye opener).* The CAGE is used as a screening test for the so-called problem drinker, suspected to be alcohol dependent, and the cutoff point adopted is two or more positive answers to the four questions. It presents high sensitivity, specificity, and predictive values, both in its English version and in the Portuguese version^14^.

Data was collected by filling out the questionnaire with sociodemographic and occupational data, followed by the application of the SRQ-20 and the CAGE test. We contacted the human resources sector of the mill to map the places of housing of the workers; thus, the instruments were applied individually in the houses or leisure spaces of the workers. For the quantitative analysis, we used the program EpiInfo for Windows, version 3.5.1. The workers were characterized based on sociodemographic and occupational data and the prevalence of the variables of CMD were described by frequency, central tendency, and dispersion measures, being presented in the tables.

Then, we carried out a cross-over between the variables and CMD to identify possible associations, using Pearson's chi-square test or Fisher's exact test. We used as a reference category, for each one of the analyzed variables, the one that present the lowest prevalence. The magnitude of the association of each variable with the presence of CMD was identified by the prevalence ratio in relation to statistical significance by the 95% confidence interval, obtained by logistic regression.

The study was approved by the Research Ethics Committee of the Centro de Ciências da Saúde of the Universidade Federal de Pernambuco (CAAE 27225414.2.0000.5208), respecting the provisions of Resolution 466/12 of the National Health Council and the declaration of Helsinki (1964). All participants signed the informed consent.

## RESULTS

A total of 110 migrant workers were studied, with a mean age of 29.6 (SD = 9.4) years. The description of the sociodemographic and occupational data is presented in Table 1.

**Table 1:** Sociodemographic and occupational characterization of the study population. (n = 110)

Variable	Category	n	%
Age (years)	18-29	69	62.7
	30-39	25	22.7
	40-49	11	10.0
	Above 50	5	4.6
Marital status	Single	53	48.2
	Married	38	34.5
	Divorced	19	17.3
Number of children	Zero	59	53.7
	Only 1	24	21.8
	2	14	12.7
	3	8	7.3
	4 or more	5	4.5
Knows how to read/write	Yes	72	65.4
	No	8	7.3
	Only signs the name	30	27.3
Education level	None	9	8.2
	Elementary school	83	75.5
	High school	18	16.3
Time cutting sugarcane (years)	1-5	59	53.6
	6-10	38	34.6
	11-15	10	9.1
	16-20	3	2.7
Family income (minimum wage)	1-2	51	46.4
	2-3	59	53.6
Type of housing	Given by the mill	98	89.1
	Rented by the workers	12	10.9
Relationship with other residents	Workers	67	60.9
	Family members	33	30.0
	Wife	10	9.1
Alcohol	Consumes	85	77.3
	Does not consume	25	22.7
CAGE	Positive	25	22.7
	Negative	85	77.3

CAGE: Cut down, Annoyed, Guilty, and Eye-opener questionnaire

In the analysis by group of symptoms (Table 2), we observed that, in the category of depressive or anxious mood, the highest number of agreement answers was for “feel unhappy”, with 72.7% of positive responses, followed by “feel nervous, tense, or worried” (59.1%).

**Table 2:** Distribution of the groups of symptoms of the SRQ-20 by affirmative answers among migrant sugarcane workers, 2014.

CMD	Question	n	%
Depressive or anxious mood	Q4 Are you easily frightened?	37	33.5
	Q6 Do you feel nervous, tense, or worried?	65	59.1
	Q9 Do you feel unhappy?	80	72.7
	Q10 Do you cry more than usual?	18	16.4
Somatic symptoms	Q1 Do you often have headaches?	57	51.8
	Q2 Is your appetite poor?	73	66.4
	Q3 Do you sleep badly?	55	50.0
	Q5 Do your hands shake?	66	60.0
	Q7 Is your digestion poor?	46	41.8
	Q19 Do you have uncomfortable feelings in your stomach?	28	25.5
Decrease in vital energy	Q8 Do you have trouble thinking clearly?	1	0.9
	Q11 Do you find it difficult to enjoy your daily activities?	0	0
	Q12 Do you find it difficult to make decisions?	38	34.5
	Q13 Is your daily work suffering?	99	90.0
	Q18 Do you feel tired all the time?	74	67.3
	Q20 Are you easily tired?	21	19.1
Depressive thoughts	Q14 Are you unable to play a useful part in life?	0	0
	Q15 Have you lost interest in things?	1	0.9
	Q16 Do you feel that you are a worthless person?	0	0
	Q17 Has the thought of ending your life been on your mind?	4	3.6

CMD: Common Mental Disorders

The overall prevalence of CMD among the workers studied was 40% (n = 44), according to SRQ-20 (cutoff score 7/8). The distribution of the prevalence of CMD by sociodemographic variables is presented in Table 3.

**Table 3:** Distribution of the prevalence of CMD by sociodemographic and occupational variables.

Characteristic		Prevalence (%)	PR	95%CI	p
Age group (years)					
	18-29	40.6	1.49	0.54-4.07	0.311
	30-39	40.0	1.47	0.50-4.31	0.366
	40-49	27.3	*	*	*
	≥ 50	60.0	2.20	0.66-7.31	0.241
Marital status					
	Single	37.7	1.19	0.57-2.52	0.634
	Married	47.4	1.50	0.71-3.15	0.259
	Divorced	31.6	*	*	*
Children					
	Yes	39.2	*	*	*
	No	40.7	0.96	0.61-1.53	0.876
Education level					
	Zero	44.4	2.67	0.75-9.45	0.139
	Elementary school	44.6	2.67	0.93-7.72	0.028
	High school	16.7	*	*	*
Number of harvests					
	1 to 5	42.4	1.27	0.25-6.47	0.621
	6 to 10	36.8	1.11	0.21-5.78	0.701
	11 to 15	40.0	1.20	0.20-7.05	0.685
	16 to 20	33.3	*	*	*
Family income					
	1-2 wages	41.2	1.06	0.67-1.67	0.815
	2-3 wages	38.9	*	*	*
Relationship with other residents					
	Other workers	36.5	*	*	*
	Family members	40.0	1.04	0.63-1.73	0.881
	Wife	50.0	1.30	0.65-2.60	0.359
Alcoholism					
	Yes	41.2	1.14	0.64-2.05	0.643
	No	36.0	*	*	*
CAGE					
	Yes	38.6	3.19	1.51-6.74	0.001
	No	12.1	*	*	*
Health-work relationship					
	Yes	50.9	1.80	1.09-2.96	0.016
	No	28.3	*	*	*
Absence from work					
	Yes	54.4	2.36	1.36-4.09	0.0008
	No	24.5	*	*	*
Medical care					
	Yes	48.5	1.78	1.03-3.06	0.026
	No	27.3	*	*	*

CMD: Common Mental Disorders; CAGE: Cut down, Annoyed, Guilty, and Eye-opener questionnaire

* Reference group.

Regarding the factors associated with CMD, workers who were positive in the evaluation of the CAGE, those who had a work-related health problem during the harvest, those who reported being absent from work, and those who received medical care during the work period presented an increased chance of being suspected of having CMD.

## DISCUSSION

All workers studied were male. The preference for male labor is linked to the new context of mechanization in sugarcane mills, as the work day and pace have been intensified, demanding from the worker physical strength and manual ability to keep pace with production and compete with the machine^15^.

The predominant age group among participants was 18-29 years (62.7%). This finding confirms what other authors^15^ ^,^ ^16^ emphasize, that is, the preference of sugarcane mills to hire young men, who have more energy and are more productive. This is explained by the fact that the work with sugarcane is a difficult task, which does not favor the inclusion of older workers. This phenomenon also justifies the reduced time of a worker's stay in sugarcane plantations – in this study, 88.2% of the workers had one to 10 years of sugarcane harvest.

According to Novaes et al.^15^, being young is also a differential. It means having a specific disposition to work, leveraged by the moment of the cycle of life when – as a rule – the search, the desire to “be someone in life” prevails, an essential motivation for young persons to face the routine and discipline at work. After all, for them, children of farmers, work is the only way to carry out personal and family projects.

Education level in the study population was another factor that deserves to be highlighted, since most (75.5%) of the subjects had only up to Elementary School – 49.1% reported not having completed it and only 26.4% reported having completed it. In addition, 27.3% of the workers “only know how to sign the name”. The low educational profile observed among migrant workers is similar to the profile found in the states of Paraíba and Pernambuco. Data from the National Household Sample Survey^c^, conducted by the IBGE in 2013, show that persons aged 25 years or more who fall into the categories “uninstructed or less than one year of study” to “eight years of study” were 64.2% in the state of Paraíba and 58.6% in the state of Pernambuco.

As a consequence of the low education level, we can observe the difficulty of insertion of these young persons in other sectors of the labor market or the lack of stability in the profession of sugarcane worker. Faced with the current process of mechanization, the worker needs to have more education and skills to handle new technologies, such as to operate harvesters and drive tractors and trucks, and to take up new jobs, such as mechanic specialized in the maintenance and repair of agricultural machinery^6^.

The monthly average income profile observed among sugarcane workers had a higher concentration (53.6%) in the stratum of two to three minimum wages (R$1,449–R$2,172), which is higher than the monthly income *(per capita)* of the states of Pernambuco and Paraíba, which is in the range of R$802.00 to R$682.00^d^.

The possibility of acquiring a higher income, which allows them to satisfy their needs while at the same time fulfill their dreams, favors the search for work in the sugarcane plantations of São Paulo, aiming, above all, to “earn money”, “buy a motorcycle”, a “house”, “have the appreciation of family members”. Knowing that young men from rural areas can hardly fulfill these dreams by working in their places of origin, the path chosen is usually that of migration to sugarcane plantations or other activities, such as construction or the harvest of coffee or orange.

Regarding the type of housing, the most reported type was the house given or leased by the mill (89.1%). According to Cover^10^, in the process of migration to sugarcane plantations, workers basically have two types of housing: houses rented by the workers themselves in the cities (in neighborhoods near the mills) in the case of migration with the family, and lodgments or houses leased by the mill to workers who migrate by themselves.

### Mental Health of Workers

The global prevalence of CMD (40%) was higher than that found in other studies that had as reference the population of rural sugarcane workers. A study carried out by Faker^a^, at a sugar and ethanol mill in the municipality of Novo Andradina, state of Mato Grosso do Sul, Brazil, has identified a prevalence of 12%. Duarte^e^, in a study carried out with sugarcane workers from the municipality of Santa Helena de Goiás, State of Goiás, Brazil, has found a prevalence of 33.1%.

The low prevalence found in the research of Faker^3^ can be explained by the fact that the population studied was not only the sugarcane workers but also mechanical cutting workers. In addition, data collection was carried out in the workspaces or paths to the sugarcane plantations and always with the presence of two employees of the technical administrative sector of the mill^a^, which may have influenced the answers of workers (information bias).

However, the findings of this study are close to other research studies carried out in rural communities and with farmers in general. In rural communities, Costa and Ludermir^17^ have found 36% of prevalence of CMD among farmers in Mata de Pernambuco, and Faria^18^ has found 38% in farmers in Serra Gaúcha, both in Brazil.

Regarding the analysis by group of symptoms of the SRQ-20, we observed that the highest concentration of positive answers (90%) was for the question “daily work suffering”. The work of manual cutting sugarcane is described by Santos^f^ as precarious work, based on the perception of the worker.

The perception of these workers about the work process and its effects on their health is that work in sugarcane plantations is hard, suffering, and harmful and this is due to the working conditions and also to the effort they are forced to make to reach the productivity averages required by the mills. This characteristic of intensified work, whose production rate is always accelerated, can bring immediate risks to the health of the worker from work accidents. Moreover, it can also generate irreversible long-term strain because the effort is continuous and the time of physical recovery and maintenance of the workforce is limited and insufficient.

Seligmann-Silva^19^ shows how physical fatigue is directly related to mental fatigue.

Mental fatigue is inseparable from physical fatigue. […] In cases in which fatigue accumulates over time, we have situations that have been named as chronic fatigue or pathological fatigue, marked not only by fatigue that does not stop after daily sleep, but also by sleep disturbances, irritability, dismay, and sometimes various pains and loss of appetite.

Dismay was also reported by the workers. In the group of depressed or anxious mood, the statement “felt sad in the last days” was very relevant among workers (72.7%). It is believed that most of these workers, because of the distance from their social and family environment, experience the drama of missing their loved ones daily.

In the group of somatic symptoms, we highlight the affirmative “poor appetite”, with 66% of affirmative answers. The lack of appetite reported by sugarcane works can also be related to the way food is prepared and stored until consumption, since we could observe that the food was prepared by the workers themselves, usually at night, being in most cases only beans, flour, rice, and meat. In addition, workers often encountered spoiled food at lunchtime.

The highest frequency of negative responses was found in the group of depressive symptoms, and the highest number of positive responses was for the affirmative “thought of ending your life”, with 3.6%.

### Factors Associated with CMD

The prevalence of CMD was higher among workers who were older than 50 years (60%), married (47.4%), who did not have children (40.7%), with one to five years of sugarcane harvest (42.4%), in the range of one to two minimum wages (41.2%), who shared the house with the wife (50%), and who consumed alcoholic beverages (41.2%). However, none of these variables presented statistical significance (p > 0.05).

Regarding the variables that were significant, workers who were positive in the evaluation of the CAGE, those who had a work-related health problem, those who reported being absent from work, and those who received medical care during the work period presented a 3.19 (p = 0.001), 1.80 (p = 0.016), 2.36 (p = 0.0008), and 1.78 (p = 0.026) times more chance of suspected CMD, respectively.

We believe that the strong association between suspected CMD and the positive evaluation of the CAGE (workers identified as problem drinkers) can lead to a disorganization of the affective investments provoked by the organizations of the work, putting in danger the mental health of the workers. According to Dejours^20^, alcoholism can be considered as a defensive strategy, used as an individual escape from anxiety related to survival and that can cause a particularly severe mental and somatic fate.

The CMD was also associated with workers who reported having been affected by a work- related health problem. Among the main illnesses described by the group of workers with suspected CMD, we can mention: cuts, spine and joint pain, skin allergy, intestinal infection, pneumonia, hypertension, urolithiasis, and labyrinthitis. All these workers were able to explain, from their experiences, the causal nexus between work in sugarcane plantations and their respective diseases.

For man, disease always corresponds to the ideology of shame of ceasing to work, as the body can only be accepted in the silence of the organs, and only the working body, the productive body of man, is accepted^21^. Such difficulty in assuming that the body is sick finds an even greater barrier when it comes to Northeastern men who were raised in an essentially *macho* culture, in which the reproduction of the idea that “true men do not get sick”, “do not feel pain”, is common. Perhaps living with the disease far from family members and closer to other men who have also been raised in this same social environment is difficult and can bring some consequence to their mental health.

Regarding absenteeism at work, we could observe that most of the workers were absent because they had a health problem. However, we also identified other reasons, such as: abuse of alcohol, separation of marriage, and courses in the driving school to have the driving exam. Any reason that causes the worker to be absent results in punishment by the mill, as it has control mechanisms to address the absentees. Workers who are often absent have their names included in a list, known as a “black list,” produced by the mill; consequently, they are unable to be hired for the next harvest^10^.

The last variable that was significant was the demand for medical care, which also results in the worker having to accept that the productive body is sick and needs care, that they need to miss work, and request a sick leave. It is known that the mill uses mechanisms to control the number of leaves^b^ and this may result in a reduction in the income or even compromise their place in the next harvest.

The results obtained in this study should be evaluated with caution because of the possible limitations of a cross-sectional study. Selection bias may be present, as we only evaluated who was working at the time of the research. Therefore, those who fell ill and lost their jobs were not part of the study (survival bias). The reverse hypothesis cannot be ruled out, i.e., we cannot identify whether the CMD influenced the associated factors or vice versa.

Another limitation was that the SRQ-20, used in data collection, was not self-administered, since most of the workers presented many difficulties related to education level. Therefore, the participation of the researcher, at this time, may have led to information bias. In addition, the number of workers who participated in the research was reduced, because, given the progress of the mechanization process and the drought phenomenon that hit the Southeast region in 2014, most workers were dismissed before the middle of the harvest. This hindered data analysis, since we obtained wide confidence intervals, which reduces the accuracy of the estimates.

## Footnotes

a Faker JN. A cana nossa de cada dia: saúde mental e qualidade de vida em trabalhadores rurais de uma usina de álcool e açúcar de Mato Grosso do Sul [dissertation]. Campo Grande: Universidade Católica Dom Bosco; 2009 [cited 2017 Jul 30]. Available from: http://site.ucdb.br/public/md-dissertacoes/8075-a-cana-nossa-de-cada-dia-saude-mental-e-qualidade-de-vida-em-trabalhadores-rurais-de-uma-usina-de-alcool-e-acucar-de-mato-grosso-do-sul.pdf

b Instituto Brasileiro de Geografia e Estatística. Brasil em Síntese: cidades e estados brasileiros. Rio de Janeiro: IBGE; s.d. [cited 2017 Sep 25]. Available from: http://www.cidades.ibge.gov.br

c Instituto Brasileiro de Geografia e Estatística. PNAD – Pesquisa Nacional por Amostra de Domicílios: síntese de indicadores sociais. Rio de Janeiro: IBGE; 2011 [cited 2017 Jul 30]. Available from: ftp://ftp.ibge.gov.br/Indicadores_Sociais/Sintese_de_Indicadores_Sociais_2014/pdf/educacao.pdf

d Instituto Brasileiro de Geografia e Estatística PNAD – Pesquisa Nacional por Amostra de Domicílios Contínua. Rio de Janeiro: IBGE; 2014 [cited 2017 Jul 30]. Available from: ftp.ibge.gov.br/Trabalho_e_Rendimento/Pesquisa_Nacional_por_Amostra_de_Domicilios_continua/Renda_domiciliar_per_capita_2014/Renda_domiciliar_per_capita_2014.pdf

e Duarte GJ. Transtornos mentais comuns em trabalhadores rurais no corte da cana-de-açúcar, Santa Helena de Goiás/Goiás [dissertation]. Goiânia: Pontifícia Universidade Católica de Goiás; 2010 [cited 2017 Jul 30]. Available from: http://tede2.pucgoias.edu.br:8080/bitstream/tede/3141/1/GUILHERME%20JOSE%20DUARTE.pdf

f Santos AP. O moinho satânico do agronegócio canavieiro no Brasil: dependência e superexploração do trabalho na região de Ribeirão Preto - SP. 2013 [thesis]. Campinas: Instituto de Filosofia e Ciências Humanas da Universidade Estadual de Campinas; 2013 [cited 2017 Jul 30]. Available from: http://repositorio.unicamp.br/jspui/bitstream/REPOSIP/280877/1/Santos_AdrianoPereira_D.pdf
